# A Unified Spatio-Temporal Inference Network for Car-Sharing Serial Prediction

**DOI:** 10.3390/s24041266

**Published:** 2024-02-16

**Authors:** Nihad Brahimi, Huaping Zhang, Syed Danial Asghar Zaidi, Lin Dai

**Affiliations:** School of Computer Science and Technology, Beijing Institute of Technology, Beijing 100081, China; brahiminihad@bit.edu.cn (N.B.); aghadani3@gmail.com (S.D.A.Z.); dailiu@bit.edu.cn (L.D.)

**Keywords:** spatio-temporal inference, prediction, temporal features, spatial feature, spatio-temporal feature, uncertainty analysis

## Abstract

Car-sharing systems require accurate demand prediction to ensure efficient resource allocation and scheduling decisions. However, developing precise predictive models for vehicle demand remains a challenging problem due to the complex spatio-temporal relationships. This paper introduces USTIN, the Unified Spatio-Temporal Inference Prediction Network, a novel neural network architecture for demand prediction. The model consists of three key components: a temporal feature unit, a spatial feature unit, and a spatio-temporal feature unit. The temporal unit utilizes historical demand data and comprises four layers, each corresponding to a different time scale (hourly, daily, weekly, and monthly). Meanwhile, the spatial unit incorporates contextual points of interest data to capture geographic demand factors around parking stations. Additionally, the spatio-temporal unit incorporates weather data to model the meteorological impacts across locations and time. We conducted extensive experiments on real-world car-sharing data. The proposed USTIN model demonstrated its ability to effectively learn intricate temporal, spatial, and spatiotemporal relationships, and outperformed existing state-of-the-art approaches. Moreover, we employed negative binomial regression with uncertainty to identify the most influential factors affecting car usage.

## 1. Introduction

Car-sharing companies have gained significant popularity in modern society due to their cost-effectiveness and convenience, providing a flexible alternative to traditional car ownership. These services alleviate various issues related to lease payments, maintenance, and parking, making them an appealing option for users seeking a hassle-free mobility solution. Beyond individual benefits, these systems contribute to reduced traffic congestion, lower carbon emissions, and minimized air pollution, positioning them as a sustainable and environmentally friendly transportation option.

However, the spatial and temporal distribution of cars across company parking stations presents a critical challenge for car-sharing firms. Accurate demand prediction is essential for optimizing resource allocation, enhancing rental rates, and improving customer satisfaction. To address these challenges, these companies leverage GPS tracking data to predict demand patterns and allocate resources effectively. These data contain a wide range of factors, such as temporal features (e.g., the average demand value in the last four time intervals), spatial features (e.g., longitude and latitude of the parking station), meteorological features (e.g., weather conditions), event features (e.g., holidays), and categories of points of interest near every station [1]. Various techniques, including predictive analytics and machine learning algorithms, aid in identifying demand trends and patterns, enabling companies to adjust their operations accordingly.

To ensure a balanced distribution of cars across various parking lots throughout the day, we propose a comprehensive Unified Spatio-Temporal Inference Prediction Network (USTIN) model. The USTIN model is a unified architecture that incorporates a temporal feature unit, a spatial feature unit, and a spatio-temporal feature unit. Leveraging a combination of Temporal Convolutional Networks (TCN), Long Short-Term Memory (LSTM), and Graph Convolutional Network (GCN), the model effectively processes and analyzes the data. Furthermore, the utilization of negative binomial regression with uncertainty has allowed for the analysis of the most influential factors affecting car usage in parking stations.

The highlights of our work include the following:

- Proposed USTIN, a unified neural architecture for car-sharing demand prediction, integrating temporal, spatial, and spatio-temporal features across multiple units.
- Achieved state-of-the-art prediction accuracy by effectively capturing complex spatial, temporal, and spatio-temporal influences on car demand.
- Identified the most influential demand factors through negative binomial regression with uncertainty to further enhance predictions.

The rest of the paper is organized as follows: Section 2 provides a literature review of the current studies on serial prediction models. In Section 3, we introduce an overview of the methods used. Section 4 details the experimental framework employed to evaluate our approach’s performance. Section 5 analyzes our prediction results. Finally, Section 6 concludes the paper and outlines potential directions for future research.

## 2. Literature Review

The objective of the traffic prediction problem is to predict future traffic flow using historical data. The key work in this area includes the DMVST-NET proposed by [1], employing local CNN and LSTM to model spatial and temporal relationships in flow. Additionally, graph deep learning techniques have gained prominence for relationship modeling within traffic networks. The authors of ref. [2] proposed a multi-graph convolutional network and an Attention-based Spatial-Temporal Graph Neural Network (ASTGNN) to model the relationships within flow networks. Similarly, the authors of ref. [3] developed a Hybrid Spatio-Temporal Graph Convolutional Network (H-STGCN) to deduce future travel time from upcoming traffic volume.

Furthermore, the challenge of predicting traffic flow is closely related to the growing need for accurate car-sharing system demand prediction [4]. Car-sharing services have exploded in popularity in recent years as an alternative mode of urban transportation. However, effectively managing these systems requires the reliable prediction of where and when vehicles will be needed. As such, many studies have begun exploring predictive models of car-sharing demand, and investigating different influencing factors. The authors of ref. [5] looked into the effects of time horizons, environmental conditions, and learning algorithm types on the prediction of vehicle availability in car-share systems. The authors of ref. [6] estimated the distance to the closest available vehicle in a fleet, whereas other researchers examined multidimensional optimization problems like station-based vehicle relocation [7,8].

Recent studies have introduced innovative models to enhance efficiency from multiple perspectives. The authors of ref. [9] compared spatially implicit Random Forest models with spatially aware methods for the spatially aware analysis of car-sharing demand. The authors of ref. [10] evaluated the use of Long Short-Term Memory (LSTM) and Prophet techniques for predicting the demand for car-sharing services. Furthermore, the authors of ref. [11] proposed a maximum entropy approach for modeling car-sharing parking dynamics.

Advancements in deep learning have shown promise in extracting spatial and temporal features for demand prediction [12]. However, effectively modeling spatial factors remains a challenge. Several studies have considered the influence of Points of Interest (POIs) near parking stations [13,14]. Notably, spatial imbalances between vehicle supply and demand have been addressed with relocation strategies [15]. Nevertheless, these models often exhibit limitations in capturing detailed spatial factors.

Despite recent strides, prior works lack multi-time scale designs to capture periodical seasonalities. Addressing this gap, the authors of refs. [16,17] introduced different timeframe durations, yet model performance diminishes in regions with varying demand densities.

This study bridges existing gaps by integrating POIs and meteorological features, taking into consideration varied time scales and addressing travel demand density. The proposed model aims to enhance the accuracy and generalization capacity, offering a holistic approach to travel demand prediction.

## 3. Methodology

### 3.1. Unified Spatio-Temporal Inference Prediction Network

**Figure 1:**
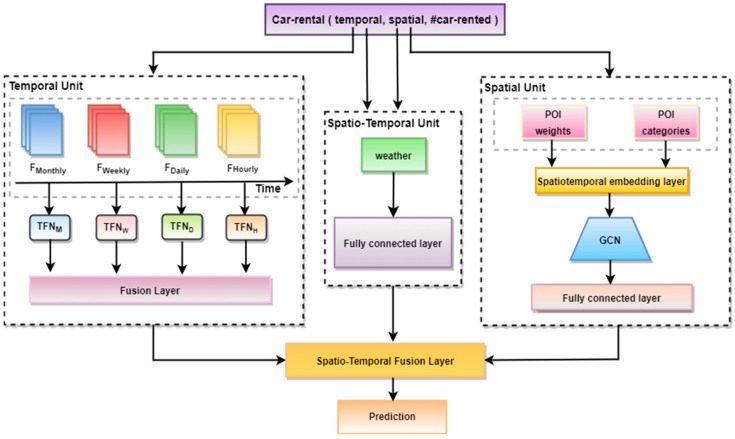
Structure of Unified Spatio-Temporal Inference Prediction Network (USTIN).

The overall architecture of the proposed Unified Spatio-Temporal Inference Prediction Network (USTIN) model is described in Figure 1. The model predicts the number of vehicles that are going to be used at a given prediction horizon.

Our approach incorporates three distinct units: a temporal feature unit, a spatial feature unit, and a spatio-temporal feature unit [18]. The different units extract key frames, enabling an accurate prediction of travel demand. The temporal unit is designed to capture temporal dependencies and comprises four layers, each corresponding to a different time scale. The spatial unit focuses on capturing spatial dependencies using Points of Interest (POIs), while the spatio-temporal unit integrates weather data to effectively capture spatio-temporal correlations. Finally, the outputs obtained from each unit are combined in the feature module fusion and training unit to generate accurate predictions of passenger demand.

#### 3.1.1. The Temporal Feature Unit

The temporal feature module contains four time scale-related layers, namely $monthly\left(F_{Monthly}\right),weekly\left(F_{Weekly}\right),daily\left(F_{Daily}\right),\;and\;hourly\left(F_{Hourly}\right)\;layers.$

**Figure 2:**
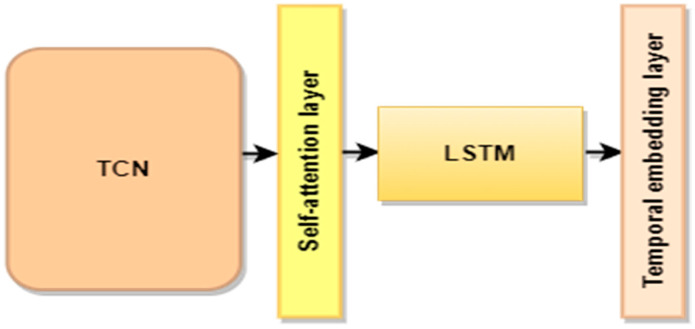
Structure of the Temporal Fusion Network (TFN).

The demand data for each layer are defined as a tensor G. Each layer corresponds to a Temporal Fusion Network (TFN) structure that effectively captures the temporal correlation, as shown in Figure 2.

1. Temporal convolutional network layer (TCN)

The tensor G is fed into a TCN layer to capture the temporal dependencies in the input data. The output $G_{t}$ is then denoted as follows:

(1) $$G_{t}=ReLU\left(W_{t}⊙G_{t-1}+b_{t}\right)$$

$W_{t}$: the weight matrix of the convolutional filter.

$b_{t}$: the bias term.

⊙: the convolution operation.

2. Self-attention mechanism layer

A self-attention mechanism is used to learn the attention weights that determine the importance of the features:

(2) $$\alpha_{t}=softmax(\frac{\left(W_{q}.G_{t}\right){\left(W_{k}.G_{t}\right)}^{T}}{\sqrt{d_{k}}})⊙G_{t}$$

$W_{q},W_{k}$: weight matrices for the query and key projections.

$d_{k}$: dimension of the key vectors.

3. Long short-term memory layer (LSTM)

The output of the self-attention mechanism enhances the LSTM’s capacity to capture temporal dependencies. This process is represented as follows:

(3) $$i_{t}=\sigma (W_{ii}\alpha_{t}+b_{ii}+W_{hi}h_{t-1}+b_{hi})$$

(4) $$f_{t}=\sigma (W_{if}\alpha_{t}+b_{if}+W_{hf}h_{t-1}+b_{hf})$$

(5) $$o_{t}=\sigma (W_{io}\alpha_{t}+b_{io}+W_{ho}h_{t-1}+b_{ho})$$

(6) $$g_{t}=tanh(W_{ig}\alpha_{t}+b_{ig}+W_{hg}h_{t-1}+b_{hg})$$

(7) $$c_{t}=i_{t}⊙g_{t}+f_{t}⊙c_{t-1}$$

(8) $$H_{t}=o_{t}⊙tanh(c_{t})$$

where

$i_{t},f_{t},o_{t},g_{t}$: input, forget, output, and candidate cell state vectors, respectively.

$W_{ii},W_{if},W_{io},W_{ig}$: weight matrices for input gate, forget gate, output gate, and candidate cell state, respectively.

$W_{hi},W_{hf},W_{ho},W_{hg}:$ weight matrices for input gate, forget gate, output gate, and candidate cell state, respectively, associated with the previous hidden state.

$b_{ii},$ $b_{if},b_{io},b_{ig}:$ bias terms for input gate, forget gate, output gate, and candidate cell state, respectively.

$c_{t}$: the cell state at time t.

$c_{t-1}$: the cell state from the previous time step.

$H_{t}$: the hidden state at time t.

σ: sigmoid activation function.

⊙: element-wise multiplication.

4. Temporal embedding layer

The temporal embedding layer is used to embed the input into a lower-dimensional space that captures the temporal relationships:

(9) $$E_{P}=ReLU\;\left(W_{t}.H_{t}+b_{t}\right)$$

$W_{t}$: weight matrix.

$b_{t}$: bias.

The four time scale-related layers are then fused. ⊗ denotes the Hadamard product, $W_{p}$, $W_{D}$, $W_{W},\;and\;W_{M}$ are the weight matrices of the time scale-related layers, and $b_{sp}$ is the bias. The output of the temporal feature module is defined according to Equation (10).

(10) $$X_{sp}=W_{p}⊗E_{p}+W_{D}⊗E_{D}+W_{W}⊗E_{W}+W_{M}⊗E_{M}+b_{sp}$$

#### 3.1.2. The Spatial Feature Unit

To effectively process Point of Interest features (POIs), we have designed a model architecture that comprises the following:

1. Spatial density calculation

POI density represents the concentration of various points of interest around every parking station. We comprehensively consider the number of POIs and the spatial distance within a Radius R.

(11) $$d\left(S_{i},{POI}_{j}\right)=2.r\;arcsin\sqrt{\sin^{2}\left(\frac{∆{lat}_{ij}}{2}\right)+\cos\left({lat}_{i}\right)\cdot \cos\left({lat}_{j}\right)\cdot \sin^{2}\left(\frac{∆{lon}_{ij}}{2}\right)}$$

$d\left(S_{i},{POI}_{j}\right)$: the distance between station $s_{i}$ and ${POI}_{j}$;

r: the radius of the Earth;

$∆{lat}_{ij}$: the difference in latitude between station $s_{i}$ and ${POI}_{j}$;

$∆{lon}_{ij}$: the difference in longitude between station $s_{i}$ and ${POI}_{j}$;

${lat}_{i}$: latitude of station $s_{i}$;

${lat}_{j}$: latitude of station ${POI}_{j}$.

The density of each POI ($D_{{POI}_{j}}$) is determined as follows:

(12) $$D_{{POI}_{j}}=\left\{\begin{array}{l} 1\;\;if\;d\left(S_{i},{POI}_{j}\right)\;\leq 1\;\;\\ 0\;\;\;\;\;\;\;\;\;\;\;\;\;\;\;\;\;\;otherwise \end{array}\right.$$

2. Regression model

The car-sharing variance is significantly greater than its average, showing an over-dispersion phenomenon [19]. Therefore, we use the negative binomial distribution to estimate the parameters. The regression model is given by the following:

(13) $$\ln\left(u_{i}\right)=\beta_{0}+\beta_{1}\cdot x_{1}+\beta_{2}\cdot x_{2}+\cdots +\beta_{n}\cdot x_{n}+\varepsilon$$

The model includes the order quantity for each car-sharing station $\left(u_{i}\right)$, the density of the POI category ($x_{1}$, …, $x_{n}$), an intercept ($\beta_{0}$), coefficients ($\beta_{1}$, …, $\beta_{n}$) for corresponding variables, and an error term (ε).

The coefficient ($\beta_{1}$, …, $\beta_{n}$) values are estimated using Maximum Likelihood Estimation (MLE), with a 5% significance level.

3. Spatiotemporal embedding layer

The tensor $G_{POI}$ and the vector $W_{POI}$, containing weights corresponding to the coefficients in Equation (13), are input into a spatiotemporal embedding layer:

(14) $$E_{POI}=ReLU\left(W_{POI}.G_{POI}\right)$$

4. Graph convolutional network layer (GCN)

The output of the spatiotemporal embedding layer is fed into a GCN layer. This layer employs the mean aggregation function to capture spatial relationships among POIs:

(15) $$H_{POI}^{n}=\left(\frac{1}{D_{POI}}\right)AGG{.E}_{POI}.A_{POI}$$

5. Fully connected layer

We used a neural network architecture with fully connected layers for feature extraction.

(16) $$X_{MC}=ReLU(W_{MC}.H_{POI}^{n}+b_{MC})$$

$W_{MC}$: weight of the fully connected layer.

$b_{MC}$: bias of the fully connected layer.

#### 3.1.3. The Spatio-Temporal Feature Unit

We used a neural network architecture with fully connected layers for the meteorological features.

(17) $$X_{ME}=ReLU(W_{ME}.G_{ME}+b_{ME})$$

$G_{ME}$: meteorological feature sensor.

$W_{ME}$: weight of the fully connected layer.

$b_{ME}$: bias of the fully connected layer.

#### 3.1.4. Feature Module Fusion and Training

The model integrates the obtained outputs via a weighted summation (Equation (18)).

(18) $$X_{ex}={W_{Sp}⊗X_{sp}+W}_{Mc}⊗X_{Mc}+W_{ME}⊗X_{ME}+b_{ex}$$

The prediction result of passenger demand ${\tilde{X}}_{tk}$ is obtained using Equation (19).

(19) $${\tilde{X}}_{tk}=W_{ex}⊗X_{ex}+b_{ex}$$

We adopt back-propagation with the Adam optimiser to improve the training efficiency [18].

### 3.2. Influential Factors Analysis

To establish the correlation between car-sharing demand and influencing factors [20], we use uncertainty estimation with coefficients ($\beta_{1}$, …, $\beta_{n}$) from the regression model (Equation (13)).

#### 3.2.1. Standard Error

The significance of the estimated values is assessed using the standard error.

(20) $$SE\left(\beta_{i}\right)=\sqrt{var\left(\beta_{i}\right)}$$

${SE(\beta }_{p})$: standard error.

${var(\beta }_{p})$: the coefficient of the corresponding variable.

#### 3.2.2. Standard Errors of Marginal Effects

To provide a measure of uncertainty, standard errors of marginal effects are associated with the marginal effects of the predictors on the response variable.

(21) $$\;\;\;\;\;\;\;\;\;\;SE\left(\frac{\partial u_{i}}{\partial x_{i}}\right)=\sqrt{{\left(\frac{\partial u_{i}}{\partial x_{i}}\right)}^{2}\sum {\left(\frac{SE\left(\beta_{i}\right)}{\beta_{i}}\right)}^{2}}$$

$\frac{\partial u_{i}}{\partial x_{i}}$: Partial derivatives of the predicted values ($u_{i}$).

The significance of the factors’ impact is defined as follows:

(22) $$Z_{i}=\frac{\frac{\partial u_{i}}{\partial x_{i}}}{SE\left(\frac{\partial u_{i}}{\partial x_{i}}\right)}$$

#### 3.2.3. *p*-Values for Marginal Effects

*p*-values for marginal effects provide insights into the significance of the factors.

(23) $${\mathrm{p-value}}_{i}=P\left(Z\geq \left|Z_{i}\right|\right)$$

Z: standard normal random variable.

$\left|Z_{i}\right|$: z-score of the i-th marginal effect.

## 4. Experiment

Section 4.1 provides an illustration of the dataset’s details, while Section 4.2 describes the experimental setting. Section 4.3 goes over the baseline models against which our model was evaluated. We describe the model configurations and the evaluation metrics in Section 4.4 and Section 4.5, respectively [21].

### 4.1. Data Description

In our study, we used the Chongqing car-sharing company’s dataset for predicting car-sharing demand, along with weather data that were acquired via web crawling [21]. Furthermore, we obtained the point-of-interest dataset via web crawling to enhance the comprehensiveness of the features used in our predictive model.

#### 4.1.1. Car-Sharing Dataset

The experiments were conducted using the pre-processed car-sharing operator dataset. The dataset contained more than 1 million records over 860 parking lots, from 1 January 2017, 00:00:00 to 31 March 2019, 23:00:00 [21].

#### 4.1.2. Weather Condition Dataset

In our work, we considered that meteorology data affected car-sharing demand [21]. Meteorology data, such as weather conditions and temperature, were collected using a Python-based Selenium web crawler to scrape the Chongqing weather condition from 1 January 2017, 00:00:00 to 31 March 2019, 23:00:00.

#### 4.1.3. Points of Interest Dataset

The car-sharing dataset was augmented with Points of Interest (POIs) data using the Baidu API for web crawling. This process involved obtaining and integrating supplementary location-based information such as restaurants, cafes, museums, cultural landmarks, and so on. The data crawling aimed to enhance the quality and diversity of the original dataset.

Table 1 presents the influencing indicator system used to determine the potential demand for car-sharing.

### 4.2. Data Pre-Processing

Raw data may contain noise, outliers, missing values, or irrelevant features, which can negatively affect the performance of machine learning models [22]. Before analysis, we applied pre-processing methods as follows:

(1) Imputation: Due to the numerical meaning of the missing values [21], we replaced them using K-nearest neighbours’ imputation.
(2) Normalization: The dataset was normalized using min-max scaling, involving scaling the numerical features to a range between 0 and 1.
(3) Clustering: The parking stations were organized into four distinct classes using frequency-based clustering [21]:
  - Class A: daily rented cars.
  - Class B: frequently used cars.
  - Class C: sometimes used cars.
  - Class D: unlike other parking stations, cars of this class are rarely used.
  Classes A, B, C, and D have different parking stations IDs, such as 16, 104, 6, and 25.
(4) Splitting the Dataset: We split the data between training and test sets. The training set starts from 1 January 2017 to 31 December 2018, and the test set from 1 January 2019 to 31 January 2019.

### 4.3. Experimental Setting

For the purpose of this study, we installed TensorFlow 1.14.0, Keras 2.2.4-tf, Pandas 0.23.4, Sklearn 0.21.1, Numpy 1.18.1, Matplotlib 3.1.0, and Statsmodels 0.10.1 [21].

The models were implemented using a PC with an i7 Intel (R) Core™i7-7500U CPU running at 3.00 GHz and 8 GB RAM with the Windows 10 operating system under the Python 3.7 development environment [23].

### 4.4. Baseline Methods

The following section outlines the baseline models against which we compared the proposed model:

(1) Multiple layer perceptron (MLP)

MLP is a feedforward neural network [24]. The network learns to map input data to the target output using backpropagation, adjusting the weights to minimize the difference between the predicted and actual outputs.

(2) K-Nearest Neighbours (KNN)

KNN works by finding the k closest neighbours; it makes predictions based on the outcome of the k neighbours closest to that point [25].

(3) Random Forest (RF)

A random forest is a collection of tree predictors [26], where each tree is generated using a random vector sampled independently from the input vector [27].

Table 2 represents the hyperparameter tuning results of the baseline models. We used the k-fold cross-validation method with k = 5 and grid search to avoid overfitting by finding the optimal hyperparameters.

(4) eXtreme Gradient Boosting (XGBoost)

XGBoost is an efficient and scalable implementation of a gradient boosting framework by (Friedman, 2001) (Friedman et al., 2000). The package includes an efficient linear model solver and tree learning algorithm [28]. XGBoost fits the new model to new residuals of the previous prediction and then minimizes the loss while adding the latest prediction [29].

(5) CNN-LSTM model

CNN-LSTM is a hybrid model built by combining CNN with LSTM for improving the accuracy of forecasting [30]. The model comprises two main components: the first component consists of convolutional and pooling layers, in which complicated mathematical operations are performed to filter the input data and extract the useful information. The second component exploits the generated features by LSTM, which possess the ability to learn long-term and short-term dependencies through the utilization of feedback connections and dense layers [31].

### 4.5. Model Configurations

We employed k-fold cross-validation with k = 5 and grid search for the hyperparameter tuning of the LSTM, TCN, and GCN models to avoid overfitting and find the optimal hyperparameters [32].

Table 3 represents the optimized hyperparameter for our proposed USTIN model.

### 4.6. Evaluation Metrics

The evaluation metrics show how well the prediction fits the past data. They help in comparing prediction techniques using the same set of data [33].

#### 4.6.1. Mean Absolute Error (MAE)

The MAE is the mean of the absolute predicted error values.

(24) $$MAE=mean\;\left(absolute\;\left({expected}_{value}-{predicted}_{value}\right)\right)$$

#### 4.6.2. Mean Square Error (MSE)

The MSE is calculated as the average of the squared predicted error values. It is well-known for putting more weight on large error values.

(25) $$MSE=mean\;\;\left({\left({expected}_{value}-{predicted}_{value}\right)}^{2}\right)$$

#### 4.6.3. Root Square Mean Error (RMSE)

RMSE penalizes large prediction errors more compared to Mean Absolute Error (MAE):

(26) RMSE=sqrt(MSE)

#### 4.6.4. Mean Absolute Percentage Error (MAPE)

The Mean Absolute Percentage Error (MAPE) is one of the most widely used measures of forecast accuracy. It can be defined by the following formula:

(27) $$MAPE=\frac{100}{n}\sum_{t=1}^{n}\left|\frac{A_{t}-F_{t}}{A_{t}}\right|$$

where $A_{t}$ is the actual value,$\;F_{t}$ is the forecast value, and n denotes the number of fitted points.

## 5. Discussion

We compared our USTIN model against several baseline models, including KNN, LSTM, RF, and MLP. Metrics such as MAE, MSE, RMSE, and MAPE are used in respective order to evaluate the results and make comparisons between our model and other state-of-the-art models.

Note that the smallest errors are shown in bold text in Table 4 and Table 5.

### 5.1. Car Usage Prediction

The main objective of this study was to build a predictive model for vehicle usage in parking lots. By predicting car usage, parking facility managers can optimize resource allocation, improve traffic flow, and enhance customer satisfaction.

#### 5.1.1. Full Data Experiment

Table 4 illustrates a performance comparison between the proposed method and baseline methods for predicting car usage in every parking station. The results show that USTIN achieves the lowest MAE (0.0308), MSE (0.1541), RMSE (0.3925), and MAPE (0.1077) among all the methods. Notably, KNN and MLP perform poorly (i.e., KNN and MLP have a MAPE of 0.5709 and 0.8874, respectively). The poor performance of the baseline models can be attributed to their failure to model the different dependencies, unlike our proposed model, which leverages temporal, spatial, and spatio-temporal information to make predictions.

#### 5.1.2. Clustered Data Experiment

We applied our model to the entire dataset, demonstrating its robust performance in predicting car-sharing demand. To further analyze the model’s performance, we also implemented our analysis in four distinct classes. For the sake of organization and not being redundant in our explanations, we only discuss the result analysis of class “A”, as other classes exhibit the same behavior and lead to the same conclusion [21].

As can be seen from Table 5—Class A, USTIN yielded the best results and had smaller evaluation errors compared to all other models. Specifically, our model achieves significant error reductions, namely (42.14%, 63.55%, 39.63%, and 52.97%) against LSTM; (44.60%, 69.52%, 44.79%, and 37.57%) against KNN; (66.18%, 69.33%, 44.62%, and 54.99%) against MLP; (43.08%, 64.15%, 40.13%, and 55.16%) against XGBOOST; (66.18%, 69.33%, 44.62%, and 54.99%) against RF; (39.45%, 60.26%, 9.85%, and 31.47%) against TCN; (50.73%, 70.60%, 16.90%, and 49.46%) against GCN; and (66.54%, 69.10%, 40.91%, and 48.27%) against CNN-LSTM.

### 5.2. Most Influential Points of Interest

The negative binomial regression with uncertainty was used to determine the key factors that impact car usage.

#### 5.2.1. Full Data Experiment

As can be seen in Table 6, numerous factors, such as the number of rented cars ($\beta_{1}$ = 0.7865, *p*-value = 0.002), workday ($\beta_{2}$= 0.4223, *p*-value = 0.005), and temperature ($\beta_{4}$ = 0.2156, *p*-value = 0.034), positively influence car-sharing demand. Conversely, rush hour ($\beta_{3}$ = −0.1523, *p*-value = 0.023) and precipitation ($\beta_{5}$= −0.0842, *p*-value = 0.046) have negative coefficients, indicating a drop in car-sharing demand during these periods. Analyzing the influence of POIs on car-sharing demand reveals that factors like domestic services (*p*-value = 0.214), beauty (*p*-value = 0.467), culture media (*p*-value = 0.165), real estate (*p*-value = 0.134), government agency (*p*-value = 0.456), entrance and exit (*p*-value = 0.145), natural features (*p*-value = 0.214), administrative landmarks (*p*-value = 0.145), and door address (*p*-value = 0.130) have *p*-values higher than 0.05, indicating their insignificant influence on car-sharing demand.

#### 5.2.2. Clustered Data Experiment

(1) Class A analysis.

Several important POIs play a crucial role in influencing the strong car-sharing demand in Table 7—Class A. Tourist attractions ($\beta_{11}$ = 0.0659, *p*-value = 0.002), education and training centres ($\beta_{14}$ = 0.0687, *p*-value = 0.007), medical facilities ($\beta_{16}$ = 0.0721, *p*-value = 0.001), finance hubs ($\beta_{19}$ = 0.0614, *p*-value = 0.001), and government agencies ($\beta_{22}$ = 0.0510, *p*-value = 0.032) exhibit a positive impact on car-sharing usage within this class. This suggests that areas with these amenities typically have greater rates of car-sharing use.

(2) Class B analysis.

Hotel and shopping amenities significantly impact demand prediction in Table 7—Class B. Their respective positive coefficients (β) (0.825, 0.0837) and respective significant *p*-values (0.011, 0.019) suggest that these main factors play a key role in car-sharing demand. In contrast, domestic services ($\beta_{9}$ = 0.0482, *p*-value = 0.315), beauty centres ($\beta_{10}$ = −0.284, *p*-value = 0.244), and tourist attractions ($\beta_{11}$ = 0.0605, *p*-value = 0.076) show no impact on the car usage rate.

(3) Class C analysis.

Hotels ($\beta_{7}$ = 0.0427, *p*-value = 0.026) and shopping centres ($\beta_{8}$ = 0.0412, *p*-value = 0.044) remain important factors, reinforcing their role as key factors in car-sharing demand in Table 7—Class C. Leisure and entertainment ($\beta_{12}$ = 0.0465, *p*-value = 0.021) also play an important role in increasing car sharing.

(4) Class D analysis.

The influential POIs in Table 7—Class D are different from those in other classes. Medical facilities ($\beta_{16}$ = 0.0285, *p*-value = 0.045) are an important factor in driving car-sharing demand, while the other factors show less impact.

### 5.3. Results Analysis

#### 5.3.1. Prediction Results

**Figure 3:**
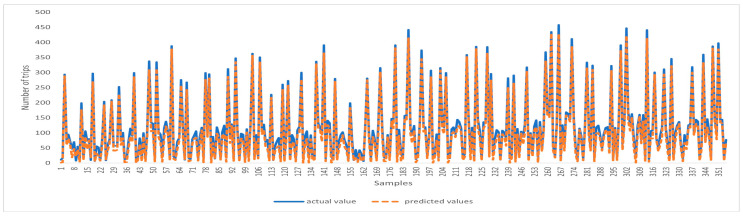
Comparison of the predicted value and the real value using USTIN—full data.

**Figure 4:**
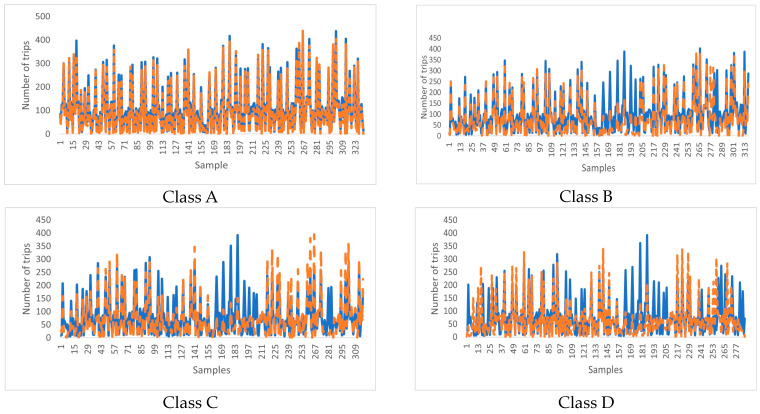
Comparison of the predicted value and the real value using USTIN—clustered data.

Figure 3 and Figure 4 present a comparison between the predicted values and the actual values obtained using the USTIN model. The results show the efficacy of the proposed neural network architecture. The integration of temporal features, spatial features, and spatio-temporal features has significantly enhanced the model’s predictive accuracy. Introducing spatial features allows the model to consider factors that are not inherently present in the spatial-temporal data but have a substantial influence on it. Furthermore, the spatio-temporal unit captures the influence of meteorological conditions across locations and time.

Overall, this study highlights the effectiveness of the proposed architecture in enhancing car-sharing demand prediction in urban environments.

#### 5.3.2. The Contribution of Influencing Factors in Car-Sharing

**Figure 5:**
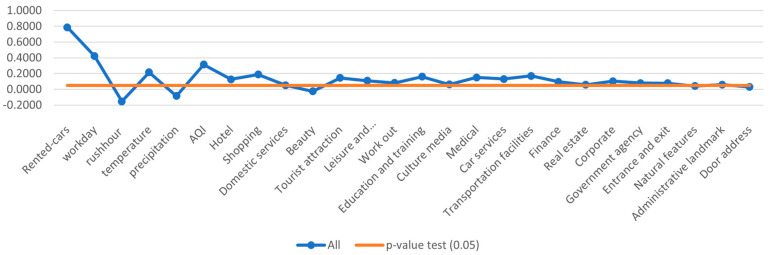
Influence of indicators on the car-sharing demand—full data.

**Figure 6:**
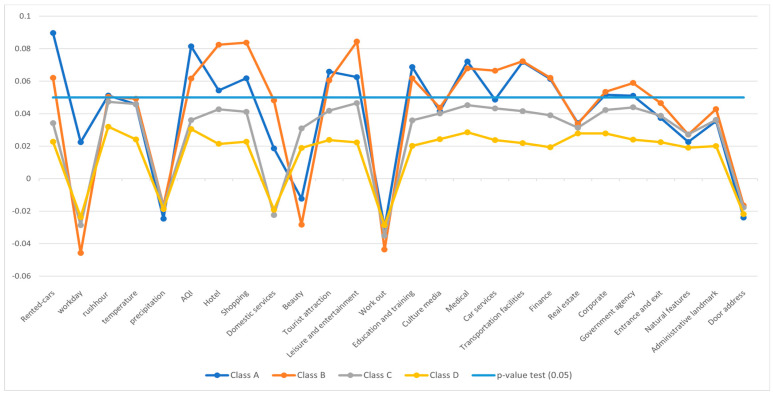
Influence of indicators on the car-sharing demand—clustered data.

Figure 5 and Figure 6 show the results of the negative binomial regression and provide insightful information on factors influencing car-sharing demand, including the number of rented cars, workday, temperature, and air quality. These factors play an important role in determining car-sharing usage. Furthermore, the evaluation results highlight the most influential Points of Interest alongside those with relatively minor impacts on car-sharing demand. Notably, tourist attractions, educational institutions, medical facilities, hotels, and shopping centers emerge as the most influential, while beauty centers, cultural landmarks, and government agencies exhibit less influence.

## 6. Conclusions

This research study has introduced the Unified Spatio-Temporal Inference Prediction Network (USTIN), an advanced architecture for predicting car usage across different parking lots. The proposed model integrates temporal, spatial, and spatio-temporal units and has demonstrated strong predictive effectiveness, outperforming other state-of-the-art models on real-world data. Notably, the temporal module adeptly captured both short- and long-term temporal demands, while the spatial module incorporates points-of-interest, enriching the contextual understanding of car usage. Additionally, the spatio-temporal module integrates meteorological data to effectively capture their influence across locations and time. Beyond car demand prediction, we used negative binomial regression with uncertainty to identify the key factors influencing car usage. The obtained results identified key drivers such as tourist destinations, hotels, and shopping centers.

While our approach offers promising results not only for online car-sharing demand prediction but also for other domains where temporal, spatial, and spatio-temporal features play a crucial role in prediction, it may exhibit some limitations in areas with very low car usage. In such regions, the model may lead to less accurate predictions. Our future research will focus on developing more advanced models that can capture the real-world complexity of spatio-temporal data. This would further enhance the efficiency of urban transportation systems and the field of spatio-temporal data analysis.

## Figures and Tables

**Table 1:** Influencing indicator system of the potential demand for car-sharing.

First-Level Indicator	Second-Level Indicator
Usage feature	$x_{1}$: Rented cars.
Temporal features	$x_{2}$: $\mathrm{workday}\;(1\;\mathrm{for}\;\mathrm{yes}\;\mathrm{and}\;0\;\mathrm{for}\;\mathrm{no}),\;x_{3}$: rushhour (1 for yes and 0 for no).
Weather conditions	$x_{4}$: $\mathrm{temperature}\;({}^{\circ}C),\;x_{5}$: $\mathrm{precipitation}\;(1\;\mathrm{for}\;\mathrm{yes}\;\mathrm{and}\;0\;\mathrm{for}\;\mathrm{no}),\;x_{6}$: AQI (Air Quality Index).
Building land attribute	$x_{7}$: $\mathrm{Hotel},\;x_{8}$: $\mathrm{Shopping}\;x_{9}$: $\mathrm{Domestic}\;\mathrm{services},\;x_{10}$: $\mathrm{Beauty},\;x_{11}$: $\mathrm{Tourist}\;\mathrm{attraction},\;x_{12}$: $\mathrm{Leisure}\;\mathrm{and}\;\mathrm{entertainment},\;x_{13}$: $\mathrm{Work}\;\mathrm{out},\;x_{14}$: $\mathrm{Education}\;\mathrm{and}\;\mathrm{training},\;x_{15}$: $\mathrm{Culture}\;\mathrm{media},\;x_{16}$: $\mathrm{Medical},\;x_{17}$: $\mathrm{Car}\;\mathrm{services},\;x_{18}$: $\mathrm{Transportation}\;\mathrm{facilities},\;x_{19}$: $\mathrm{Finance},\;x_{20}$: $\mathrm{Real}\;\mathrm{estate},\;x_{21}$: $\mathrm{Corporate},\;x_{22}$: $\mathrm{Government}\;\mathrm{agency},\;x_{23}$: $\mathrm{Entrance}\;\mathrm{and}\;\mathrm{exit},\;x_{24}$: $\mathrm{Natural}\;\mathrm{features},\;x_{25}$: $\mathrm{Administrative}\;\mathrm{landmark},\;x_{26}$: Door address.

**Table 2:** Hyperparameters for baseline models.

Model	Hyperparameters
MLP	- 2 fully connected layers, 20 and 15 hidden units.
XGBoost	- N_estimators: 25. - Max_depth: 5.
KNN	- N_neighbours: 5. - Weights: “uniform”.
RF	- N_estimators: 100. - Max_depth: 5. - Min_samples_split: 15.
LSTM	- Hidden layers: 2. - Hidden units: 25, 15 neurons. - Learning rate: 0.01. - Drop out: 0.5. - Optimizer: Adam. - Epochs: 80.
TCN	- Hidden layers: 3. - Kernel size: 3. - Dilations: [1, 2, 4, 8, 16, 32, 64]. - Number filters: 64. - Learning rate: 0.01.
GCN	- Hidden layers: 2. - Hidden units: 32, 64 neurons. - Learning rate: 0.01.
CNN-LSTM	- Convolutional Layer: 1. - Kernel Size: 5. - Filters: 64. - Max Pooling Layer: 1. - LSTM: 1 (15 neurons). - Dense Layer: 32Neurons. - Dropout 0.3. - Epochs: 100.

**Table 3:** Hyperparameters for USTIN.

Model	Hyperparameters
TCN	- Hidden layers: 3. - Kernel size: 3. - Dilations: [1, 2, 4, 8, 16, 32, 64]. - Number filters: 64. - Learning rate: 0.01. - Drop out: 0.2. - Optimizer: Adam. - Epochs: 80.
LSTM	- Hidden layers: 2. - Hidden units: 25, 15 neurons. - Learning rate: 0.01. - Drop out: 0.3. - Optimizer: Adam. - Epochs: 100.
GCN	- Hidden layers: 2 (32, 64 neurons). - Hidden units: 32, 64 neurons. - Learning rate: 0.01. - Epochs: 80.

**Table 4:** Evaluation results—full data.

	MAE	MSE	RMSE	MAPE
**KNN**	0.6012	0.5157	0.7181	0.5709
**LSTM**	0.1350	0.3303	0.5747	0.1399
**TCN**	0.1452	0.1689	0.4110	0.1097
**GCN**	0.0475	0.1825	0.4272	0.1950
**CNN-LSTM**	0.0325	0.1750	0.4183	0.1150
**RF**	0.1779	0.3561	0.5967	0.4685
**MLP**	0.6266	0.5543	0.7445	0.8874
**XGBoost**	0.0769	0. 1677	0.4095	0.1648
**USTIN**	**0.0308**	**0.1541**	**0.3925**	**0.1077**

**Table 5:** Evaluation results—clustered data.

	MAE	MSE	RMSE	MAPE
**KNN**	0.1900	0.3223	0.5686	0.2411
**LSTM**	0.1177	0.3150	0.5612	**0.2130**
**TCN**	0.2892	0.2642	0.5140	0.2924
**GCN**	0.2348	0.2845	0.5334	0.3354
**CNN-LSTM**	0.3432	0.3042	0.5515	0.5042
**RF**	0.7634	0.4106	0.6407	0.2374
**MLP**	0.3291	0.2591	0.5090	0.3498
**XGBoost**	0.3042	0.2321	0.4818	0.2213
**USTIN**	**0.1154**	**0.1054**	**0.3246**	0.2209
(a) Class A				
**KNN**	0.1956	0.3458	0.5880	0.3592
**LSTM**	0.1873	0.2892	0.5378	0.4768
**TCN**	0.2242	0.2084	0.4565	0.3424
**GCN**	0.2902	0.3875	0.6225	0.3835
**CNN-LSTM**	0.3452	0.3702	0.6084	0.5186
**RF**	0.3205	0.3437	0.5863	0.4982
**MLP**	0.1957	0.3133	0.5597	0.4234
**XGBoost**	0.1904	0.2941	0.5423	0.5002
**USTIN**	**0.1084**	**0.1912**	**0.4373**	**0.1798**
(b) Class B				
**KNN**	0.1266	0.3328	0.5769	0.2950
**LSTM**	0.1370	0.2341	0.4839	0.2705
**TCN**	0.2802	0.3842	0.6198	0.3414
**GCN**	0.2702	0.3424	0.5851	0.3845
**CNN-LSTM**	0.2441	0.3744	0.6119	0.4131
**RF**	0.8391	0.9622	0.9809	0.2242
**MLP**	0.3144	0.2538	0.5038	0.2291
**XGBoost**	0.3050	0.2588	0.5087	0.2275
**USTIN**	**0.1210**	**0.2071**	**0.4551**	**0.2242**
(c) Class C				
**KNN**	0.1294	0.2530	0.5030	0.3345
**LSTM**	**0.1185**	0.2347	0.4844	0.3802
**TCN**	0.2846	0.4242	0.6513	0.4204
**GCN**	0.3027	0.3904	0.6248	0.4684
**CNN-LSTM**	0.2221	0.3698	0.6081	0.4548
**RF**	0.6132	0.3495	0.5912	0.4863
**MLP**	0.3136	0.2538	0.5038	**0.2283**
**XGBoost**	0.3216	0.2596	0.5095	0.2468
**USTIN**	0.1885	**0.2321**	**0.4818**	0.4982
(d) Class D				

**Table 6:** Evaluation of negative binomial regression model—full data.

Parameter	Coef.	*p*-Value	Sig.	Parameter	Coef.	*p*-Value	Sig.
$\beta_{0}$	0.2845	/	/	$\beta_{14}$	0.1609	0.002	Yes
$\beta_{1}$	0.7865	0.002	Yes	$\beta_{15}$	0.0623	0.165	No
$\beta_{2}$	0.4223	0.005	Yes	$\beta_{16}$	0.1508	0.013	Yes
$\beta_{3}$	−0.1523	0.023	Yes	$\beta_{17}$	0.1303	0.006	Yes
$\beta_{4}$	0.2156	0.034	Yes	$\beta_{18}$	0.1712	0.001	Yes
$\beta_{5}$	−0.0842	0.046	Yes	$\beta_{19}$	0.0959	0.025	Yes
$\beta_{6}$	0.3156	0.063	No	$\beta_{20}$	0.0587	0.134	No
$\beta_{7}$	0.1265	0.012	Yes	$\beta_{21}$	0.1041	0.064	Yes
$\beta_{8}$	0.1889	0.005	Yes	$\beta_{22}$	0.0801	0.456	No
$\beta_{9}$	0.0523	0.214	No	$\beta_{23}$	0.0772	0.145	No
$\beta_{10}$	−0.0252	0.467	No	$\beta_{24}$	0.0433	0.214	No
$\beta_{11}$	0.1459	0.001	Yes	$\beta_{25}$	0.0613	0.145	No
$\beta_{12}$	0.1103	0.006	Yes	$\beta_{26}$	0.0303	0.130	No
$\beta_{13}$	0.0823	0.012	Yes				

**Table 7:** Evaluation of negative binomial regression model—clustered data.

Parameter	Coef.	*p*-Value	Sig.	Parameter	Coef.	*p*-Value	Sig.
$\beta_{0}$	0.2349	/	/	$\beta_{14}$	0.0687	0.007	Yes
$\beta_{1}$	0.0897	0.011	Yes	$\beta_{15}$	0.0415	0.475	No
$\beta_{2}$	0.0224	0.027	Yes	$\beta_{16}$	0.0721	0.001	Yes
$\beta_{3}$	0.0512	0.042	Yes	$\beta_{17}$	0.0486	0.214	No
$\beta_{4}$	0.0458	0.036	Yes	$\beta_{18}$	0.0719	0.049	Yes
$\beta_{5}$	−0.0246	0.118	No	$\beta_{19}$	0.0614	0.001	Yes
$\beta_{6}$	0.0815	0.159	No	$\beta_{20}$	0.0342	0.083	No
$\beta_{7}$	0.0543	0.006	Yes	$\beta_{21}$	0.0516	0.024	yes
$\beta_{8}$	0.0618	0.026	Yes	$\beta_{22}$	0.0510	0.032	Yes
$\beta_{9}$	0.0187	0.270	No	$\beta_{23}$	0.0373	0.017	Yes
$\beta_{10}$	−0.0124	0.548	No	$\beta_{24}$	0.0226	0.081	No
$\beta_{11}$	0.0659	0.002	Yes	$\beta_{25}$	0.0355	0.124	No
$\beta_{12}$	0.0625	0.221	No	$\beta_{26}$	−0.0239	0.165	No
$\beta_{13}$	−0.0301	0.074	No				
(a) Class A							
$\beta_{0}$	0.1233	/	/	$\beta_{14}$	0.0618	0.077	No
$\beta_{1}$	0.0621	0.031	Yes	$\beta_{15}$	0.0437	0.152	No
$\beta_{2}$	−0.0458	0.021	Yes	$\beta_{16}$	0.0679	0.005	Yes
$\beta_{3}$	0.0503	0.045	Yes	$\beta_{17}$	0.0665	0.391	No
$\beta_{4}$	0.0492	0.047	Yes	$\beta_{18}$	0.0723	0.020	Yes
$\beta_{5}$	−0.0168	0.011	Yes	$\beta_{19}$	0.0621	0.018	Yes
$\beta_{6}$	0.0616	0.004	Yes	$\beta_{20}$	0.0336	0.047	Yes
$\beta_{7}$	0.0825	0.011	Yes	$\beta_{21}$	0.0534	0.042	Yes
$\beta_{8}$	0.0837	0.019	Yes	$\beta_{22}$	0.0589	0.095	No
$\beta_{9}$	0.0482	0.315	No	$\beta_{23}$	0.0465	0.076	No
$\beta_{10}$	−0.0284	0.244	No	$\beta_{24}$	0.0271	0.268	No
$\beta_{11}$	0.0605	0.076	No	$\beta_{25}$	0.0429	0.155	No
$\beta_{12}$	0.0844	0.113	No	$\beta_{26}$	−0.0165	0.409	No
$\beta_{13}$	−0.0436	0.213	No				
(b) Class B							
$\beta_{0}$	0.3067	/	/	$\beta_{14}$	0.0359	0.017	Yes
$\beta_{1}$	0.0342	0.023	Yes	$\beta_{15}$	0.0401	0.295	No
$\beta_{2}$	−0.0287	0.042	Yes	$\beta_{16}$	0.0453	0.076	No
$\beta_{3}$	0.0473	0.049	Yes	$\beta_{17}$	0.0432	0.547	No
$\beta_{4}$	0.0459	0.035	Yes	$\beta_{18}$	0.0416	0.487	No
$\beta_{5}$	−0.0172	0.029	Yes	$\beta_{19}$	0.0390	0.176	No
$\beta_{6}$	0.0361	0.189	No	$\beta_{20}$	0.0314	0.192	No
$\beta_{7}$	0.0427	0.026	Yes	$\beta_{21}$	0.0423	0.077	No
$\beta_{8}$	0.0412	0.044	Yes	$\beta_{22}$	0.0439	0.172	No
$\beta_{9}$	−0.0224	0.223	No	$\beta_{23}$	0.0387	0.121	No
$\beta_{10}$	0.0310	0.709	No	$\beta_{24}$	0.0273	0.921	No
$\beta_{11}$	0.0418	0.412	No	$\beta_{25}$	0.0362	0.522	No
$\beta_{12}$	0.0465	0.021	Yes	$\beta_{26}$	−0.0178	0.123	No
$\beta_{13}$	−0.0351	0.498	No				
(c) Class C							
$\beta_{0}$	0.4387	/	/	$\beta_{14}$	0.0202	0.065	No
$\beta_{1}$	0.0228	0.021	Yes	$\beta_{15}$	0.0243	0.530	No
$\beta_{2}$	−0.0237	0.016	Yes	$\beta_{16}$	0.0285	0.045	Yes
$\beta_{3}$	0.0320	0.040	Yes	$\beta_{17}$	0.0237	0.081	No
$\beta_{4}$	0.0242	0.026	Yes	$\beta_{18}$	0.0219	0.048	No
$\beta_{5}$	−0.0187	0.013	Yes	$\beta_{19}$	0.0193	0.464	No
$\beta_{6}$	0.0305	0.726	No	$\beta_{20}$	0.0278	0.369	No
$\beta_{7}$	0.0214	0.089	No	$\beta_{21}$	0.0278	0.387	No
$\beta_{8}$	0.0227	0.020	Yes	$\beta_{22}$	0.0240	0.180	No
$\beta_{9}$	−0.0191	0.414	No	$\beta_{23}$	0.0225	0.059	No
$\beta_{10}$	0.0189	0.150	No	$\beta_{24}$	0.0190	0.837	No
$\beta_{11}$	0.0238	0.132	No	$\beta_{25}$	0.0201	0.058	No
$\beta_{12}$	0.0223	0.373	No	$\beta_{26}$	−0.0218	0.247	No
$\beta_{13}$	−0.0287	0.423	No				
(d) Class D							

## Data Availability

The data used to support the findings of this study are available from the corresponding author upon request.
